# Eye movements as a predictor of preference for progressive power lenses

**DOI:** 10.16910/jemr.15.2.6

**Published:** 2022-06-30

**Authors:** Pablo Concepcion-Grande, Amelia González, Eva Chamorro, José Miguel Cleva, José Alonso, Jose Antonio Gómez-Pedrero

**Affiliations:** Clinical research department, Indizen Optical Technologies, Spain; Applied Optics Complutense Group, Optics Department, Optics and Optometry Faculty, Complutense University of Madrid, Spain; These authors contributed equally to this work

**Keywords:** eye tracking, complete fixation time, fixation duration mean, progressive power lenses, visual perception

## Abstract

The purpose of this study is to determine if there is any correlation between the characteristics of the user’s eye movements (EMs) and the preference of the user when wearing different Progressive power lenses (PPLs) distributions. An eye-tracker system with a sample rate of 120Hz and temporal resolution of 8.3ms (Tobii-X3-120) was used to register EMs of 38 PPL users when reading in a computer screen with 2 types of PPLs (PPLsoft and PPL-hard). Number of fixations, complete fixation time, fixation duration mean, saccade duration mean, saccade distance mean, and number of regressions were analyzed for 6 different regions of the computer screen. A statistically significant difference was observed between the characteristics of the user’s EMs and the user’s PPL subjective preference (p < 0.05*). Subjects that preferred the PPL-hard presented significantly lower complete fixation time, lower fixation duration mean and lower number of regressions than those subjects indicating a preference for the PPL-soft. Results of this study suggest that eye-tracking systems can be used as PPL design recommendation systems according to the user EMs performance.

## Introduction

Presbyopia is the last stage of a gradual loss of accommodative amplitude due to the loss of elasticity of the crystalline lens. Accommodation amplitude starts to decrease from childhood, but it is around the age of forty when difficulties performing tasks at near vision start. At this age, the accommodative amplitude is between 3-4D, which is not enough to maintain clear and comfortable near vision. This decline continues into the mid-fifties when accommodation reaches its minimum value (29).

Progressive power ophthalmic lenses (PPL) are one of the most common solutions for presbyopia. A PPL is a multifocal lens that provides a continuously smooth increase in spherical power from the upper part of the lens to the lower portion, allowing the users to see clearly at all distances by changing the gaze position (31). Nevertheless, the resulting lens geometry leads to unwanted lateral astigmatism, which limits the undistorted visual areas of the users (37). The unwanted power variation in the peripheral regions of the lens is responsible for unwanted distortion, swim effect and blurriness when viewing through these regions (16).

Recent advances in the manufacturing processes of PPLs using Free-Form technology have provided a wide variety of PPL designs to the ophthalmic industry (1). This technology is currently used in conjunction with optical design software to minimize the oblique aberrations for all gaze directions by computing customized surfaces. Therefore, from a spectacles dispensing perspective, clinicians and practitioners need methods that allow the evaluation of the PPL properties according to the power distribution of the lens design. This means that it is important to know the main characteristics of the design to correctly select the PPL that best fits the user needs. To help in this selection it is important to have tools to evaluate a PPL. Some proposed methods are based on the representation of theoretical power maps of the lenses, either obtained with lens mappers (35, 36) or computed by exact ray tracing to provide user-perceived power maps (4, 5) giving a better simulation of the lens design. These analyses are based on geometrical magnitudes calculations that estimates the theoretical undistorted viewing area for each visual area of the lens. The main limitation of these methods is that the correlation between the proposed theoretical magnitudes and the PPL optical performance remains unknown.

Although it is possible to characterize a PPL by means of power distribution maps, the selection of the lens that best fits the user needs is complicated. Undistorted viewing areas calculated theoretically through the power distribution maps, do not represent the visual perception while wearing a PPL, that varies for every subject. So, in order to better understand the visual perception provided by a PPL, previous works have tried to characterize the performance of PPLs using visual tests as visual acuity (6; 7), contrast sensitivity (34), reading performance (33; 34), adaptation to skew distortion (15), and other measurements of optometric and/or ergonomic parameters (2, 3; 28; 34). Other works paid considerable attention to analyzing the relationship between the use of PPLs and the changes of the statistical descriptors of head and eye movements (EMs) when conducting visual tasks (32). In this regard, eye tracking techniques have shown some good results as tools for the characterization of optical and head movements of the user and the possible correlation with lens performance (9, 8; 17; 18; 23; 28; 34). But these methods are not completely helpful to assist in the selection of the design which better fits the user because they do not correlate specific characteristics of the users with the user satisfaction with the lens. In fact, the few studies evaluating user preference of PPL are based on subjective questionnaires (19; 39).

As indicated earlier, the optical properties of PPLs have some implications in terms of ergonomics, mainly at mid-range and near vision. The use of PPLs and the position of visual displays such as monitor screens or laptops, can have musculoskeletal or visual implications (28). In those situations, PPLs have two handicaps due to the power distribution. Horizontally, clear vision is restricted to the central region of the lens and vertically clear vision is limited to a small region related to the lens power needed for the working distance. Thus, from an ergonomic perspective it is important to find the power distribution which provides a better ergonomic position, specially at mid-range vision and near distances (35, 36; 37; 38).

Video-based eye trackers monitor eye position by video recording the reflection of an infra-red light projected in the subject’s eye (10; 21). Eye-tracking is becoming more common in a variety of applications, including mobile phones, cars, marketing, education, video games, among others (11; 13; 14; 24; 25; 26). In the research field, eye-trackers are increasingly a requirement for controlling where subjects look while performing different tasks (12; 20; 22; 30). Y. Han et al. used eye-tracking techniques to measure EMs while wearing either progressive or single-vision lenses, and while performing different visual tasks (17; 18). Concepción et al. evaluated the visual behavior in a group of 20 presbyopic participants when they were reading a text on a computer screen, concluding that EMs change when the subjects use PPLs (8). Rifai et al. evaluated head and gaze movements in a group of subjects during driving. Measurements were done in 17 PPL-wearers and 27 non-PPL wearers. Results showed that eye-head coordination was strengthened in PPLs wearers by an increase in head gain (32).

In the beforementioned studies by Y. Han et al. and Hutchings et al. significant differences in some statistical descriptors of the EMs were found when comparing PPLs and single-vision lenses, but no differences were detected when comparing different PPL designs (17; 18; 23). So, our main goal was to explore the potential of use of the eye tracker in the evaluation of PPL performance and possible determination of the best PPL for the user according to some objective parameters. For that reason, a study has been conducted to determine if the user preference for a specific power distribution design is associated to the different reading patterns with the hypothesis that those Subjects habituated to softer PPL designs, or a poorer visual quality are more tolerant to low amounts of blur through longer fixations. Then fixation duration could be used as a measure of blur tolerance, and thus an indicator of PPL softness preference.

## Methods

### Design

A prospective observational longitudinal double-blind study following the tenets of the Declaration of Helsinki was carried out. Full approval for the study was obtained from the Hospital Clínico San Carlos Ethics Committee’s (CEIC) Review Board. All participants provided their written informed consent and at the end of the trial participants were compensated with two pair of spectacles.

### Participants

The study sample comprised presbyopic subjects of both genders aged over 42 years with the following inclusion criteria: 1) Refractive error between -6.00D and +4.00D with an astigmatism lower or equal to 2.50D and addition between +1.00D and +3.00D. 2) Best corrected VA better than 0.05logMAR binocularly and 0.10logMAR monocularly. 3) Difference in refractive error between both eyes lower than 1.50D. Subjects were excluded if they had significant binocular vision anomalies, ocular pathologies (glaucoma, retinopathies, etc.) or if they had been in any pharmacological treatment that could affect the visual function.

### Procedure

The study required 4 visits. The recruitment visit (visit 1) consisted in a screening visit where refraction, selection of frames and signing of the consent form were done. In this visit, all subjects underwent a complete visual exam to check whether they meet the inclusion/exclusion criteria. The visual exam was composed by VA testing, binocular refraction, stereoacuity using Titmus test Stereo fly test, Stereoptical CO, USA), Worth test, cover test and binocular motility. Once the optometrist confirmed the participant fulfilled the inclusion criteria, the subject selected a frame model that was adapted prior to the measurements of the fitting and position-of-wear parameters. The pupillary distance was measured using an automated pupillometer, segment height was measured manually and the additional fitting parameters, which included pantoscopic tilt, back vertex distance and frame wrap angle, were measured using a special ruler (Personalization Key, IOT, Spain). At visit 2, optometrists gave instructions to participants about how to use the new progressive lenses. During this visit, the first pair of lenses (according to the randomization assignment) was dispensed. After using the lenses for 7 days through the whole day, a register of EMs using eye-tracking was carried out in visit 3. Also, assessment questionnaires were collected, and the subjects were provided with a second pair of spectacles. At visit 4, the same sequence of evaluation was conducted for the second pair of spectacles after 7 days of wear.

### Materials

#### Progressive Power Lenses

**Figure 1.**
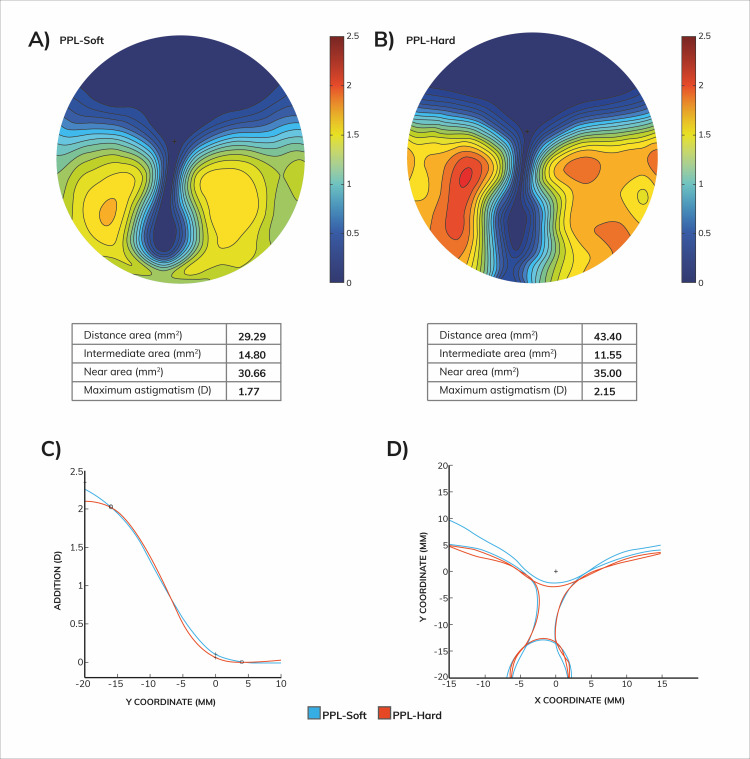
Power distribution maps of the PPL designs tested in this study. A) Cylinder power distribution maps and visual area according to Sheedy criteria of the PPL-soft lens. B) Cylinder power distribution maps and visual area according to Sheedy criteria of the PPL-hard lens. C) Progression profile. D) Sheedy’s contours (36; 37).

Two free-form PPL designs, soft and hard, were developed by IOT (Madrid, Spain) for this research. Cylinder maps and progression power profiles (as perceived by the user) are shown in Figure 1. PPL-soft is a softer overall design, meaning that the gradient of the power distribution is smaller when compared to PPL-hard. The power profiles are quite similar in both lenses, but PPL-soft has a smoother start of the progression, smaller slope, and a non-stabilized profile end. Peripheral astigmatism is spread over a wider area at the nasal and temporal sides of PPL-soft. These features translate into a smoother cylinder distribution, with smaller values of the maximum of astigmatism. As expected, the improved smoothness of PPL-soft comes at the cost of narrower far and near fields of view. The theoretical areas for the different undistorted viewing areas according to Sheedy's criteria (36; 37) are provided for the two designs in the tables included in Figure 1. For each subject, both designs were manufactured with identical prescriptions, monocular pupillary distances and pupil heights, frame parameters, lens materials (index 1.6) and anti-reflective coating. Lenses were marked with invisible laser marks with alphanumeric codes that were unmasked after the full data collection. Both pairs of spectacles were verified upon receipt (power, mounting and fitting terms) according to ISO tolerances in all surface points using a Dual Lens Mapper (Automation & Robotics, Verviers, Belgium) (27).

### Eye-tracking recording

Binocular eye position was registered when participants were reading a text in a computer screen when using the two different PPLs. An eye tracker Tobii-Pro-X3-120 (Tobii AB, Sweden) was used to record the horizontal and vertical EMs with a sample rate of 120 Hz and temporal resolution of 8.3ms, then data were processed using an open-source software designed for the analysis of EMs called OGAMA (Berlin, Germany) (40). Relative height of the screen was the same for all subjects for primary gaze position. This alignment was made by adjusting the vertical position of the screen using an adjustable table. After the vertical positioning of the screen a binocular calibration with 5 dots was made. Dots were equally spaced, 4 of them were located on the corners of the screen and the other in the center of the screen. Once calibration was complete, the reading text was showed on the screen and the registration of the EMs was made. The EM parameters analyzed were fixations (complete fixation time, number of fixations and fixation duration mean), saccades (saccade distance mean and saccade duration mean) and number of regressions (go-back fixations to re-read the text). To determine the EMs parameters, it was applied a fixation detection algorithm developed by LC technologies (LC Technologies. (2006). Fixation Functions Source Code. Fairfax, Virginia, USA: LC Technologies) using the following criteria to determine a fixation: maximum distance in pixels that a point may vary from the average fixation point and still be considered part of the fixation of 0.32°; a minimum number of samples that can be considered a fixation of 10; a fixation detection ring size of 0.49°; and merging consecutive fixations within max distance into one fixation. All these parameters were registered without chinrest, so the subject can move the head with freedom allowing them a comfortable reading to evaluate the PPLs performance in a normal use of them. Working distance was measured manually before and after each recording to guarantee it was the same for all subjects and recordings. All recordings followed a two-step data quality assessment to guarantee the good quality of the data. First a manual visual inspection of the data was carried out to ensure there are no offsets or artefacts in the data sample. The second step of the quality assessment consisted in a data loss calculation before applying any noise reduction filter. To classify a recording as having good quality for the analysis, it must be correct according to the visual inspection and it must have a percentage of data loss less than 10% before applying any noise reduction filter.

### Reading text

**Figure 2.**
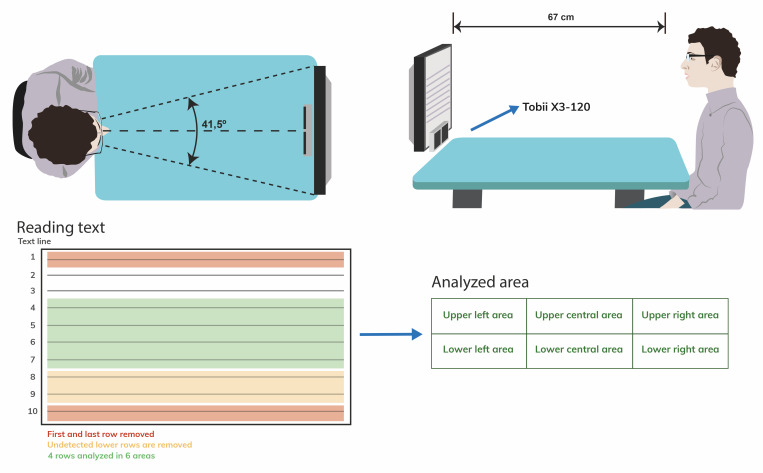
Scheme of the evaluation of EMs using eye tracking technology. Characteristics of the reading test indicating the different reading areas analyzed (rows and columns).

Participants were asked to read 2 texts composed by 10 lines with VA=0.5 logMAR located 67cm away from the participant’s eye, therefore subtending a horizontal viewing angle of 41.5º. Different reading tests with similar difficulty level were used for the evaluation of PPL-soft and PPL-hard (similar number of words, syllables and punctation marks) in a randomized order. For statistical analysis, it was selected the lowest 4 reading rows recorded by the eye-tracker device, except for the first and last rows that were removed for all subjects to avoid EMs errors when subject reach the start and end of the reading test. To analyze differences in EMs between reading areas, the text was divided in three vertical regions (two lateral parts and the central part) with the same width and two horizontal regions (upper and lower) formed by two text rows each as shown in Figure 2.

### Subjective Evaluation

Subjects were asked to use both pair of spectacles for 7 days each in their daily activities. After using the lenses, a double-blind comparison was carried out for mid-range vision. For this purpose, subjects were asked to change the spectacles when they were looking a text on a computer screen and choose the PPL which provides them a better visual quality and comfort. According to their preference, subjects were divided into 2 groups: Group 1 including those subjects that preferred the design PPL-Soft and group 2 comprised by those subjects that preferred the lens PPL-hard.

### Statistical Analysis

All statistical tests were done using Statgraphics Cen-turion XVI.II Software. The level of significance was set at 0.05. Statistical Power was set at 0.8. Multifactorial ANOVA was used to determine differences in EM pattern depending on the user’s PPL preference. Design of randomized complete block test was used to determine the relationship between the reading region and the EMs for each PPL. Finally, regarding the comparison between naïve subjects and experienced subjects a design of randomized block test was used.

## Results

### Sample characteristics

Recordings of 49 subjects were collected for both pair of spectacles, 11 of them were discarded due to data quality was not within the data quality criteria. The final sample was comprised of 38 subjects of mean age 54±5 (44-64 years), 22 of them were experienced PPL subjects and 16 were naïve. Experienced users are those subjects who had been using PPL at least 6 months before the study was carried out while naïve users are those subjects who have never used PPL. According to the subjective evaluation, there were 21 subjects that preferred the PPL-soft (Group 1) and 17 participants whose preferred lens design was PPL-hard (Group 2). Table 1 shows the prescription distribution of all participants.

**Table 1.** Refractive error, addition, type of user and preference distribution for each participant.

ID	Right eye			Left eye			Addition	User	Preference
	Sphere	Cylinder	Axis	Sphere	Cylinder	Axis			
1	2	-1.25	95	1.5	-0.5	70	2.25	Experienced user	PPL-Hard
2	1.25	-1.25	10	1	-1.25	160	2.25	Experienced user	PPL-Soft
3	2.75	-0.75	15	2.75	-0.5	175	2	Experienced user	PPL-Soft
4	2	-0.5	180	0.75	-0.5	10	2	Experienced user	PPL-Soft
5	-1	0	0	-0.5	-0.5	165	2	Experienced user	PPL-Hard
6	-4.5	-0.5	160	-4.25	-1.25	180	2.25	Experienced user	PPL-Hard
7	1.5	-0.5	30	1.5	0	0	2	Experienced user	PPL-Soft
8	0.75	-0.25	15	0.75	0	0	1.75	Experienced user	PPL-Hard
9	0	-1.75	180	0	-1.5	180	2	Experienced user	PPL-Hard
10	-1.25	0	0	-1.25	-0.5	85	1.75	Naïve user	PPL-Soft
11	0	-0.5	110	0.5	-0.25	35	1.75	Experienced user	PPL-Hard
12	0.25	-0.25	165	0	0	0	2.25	Experienced user	PPL-Hard
13	1.75	-0.5	120	1.75	-0.5	70	2.25	Experienced user	PPL-Hard
14	0.75	-0.5	85	0.75	-0.5	85	2.25	Naïve user	PPL-Soft
15	-0.5	-2.25	120	-0.25	-2.25	45	2.25	Naïve user	PPL-Hard
16	1.25	-0.5	80	-0.75	-0.5	180	2.75	Experienced user	PPL-Soft
17	-1.5	-1.5	5	-2	-1.75	160	2	Experienced user	PPL-Soft
18	2	0	0	2.25	0	0	2	Experienced user	PPL-Soft
19	5.25	-1.5	5	5.5	-1.25	155	1	Naïve user	PPL-Soft
20	1	0	0	0.75	0	0	1.75	Experienced user	PPL-Hard
21	-0.5	0	0	-0.5	-0.5	70	2	Naïve user	PPL-Soft
22	-0.5	-1.25	170	-1.75	0	0	1.25	Naïve user	PPL-Hard
23	-2.25	-1.75	90	-3	-0.5	75	1.25	Naïve user	PPL-Soft
24	-1.75	-0.75	5	-1.75	-0.75	160	1.25	Experienced user	PPL-Hard
25	0	-0.25	170	0.5	-0.75	15	1	Naïve user	PPL-Soft
26	-3.75	-0.25	55	-4	-0.25	130	0.75	Naïve user	PPL-Hard
27	0	-0.5	80	0	-0.75	80	1.75	Naïve user	PPL-Hard
28	1.25	-1	15	0.75	-0.75	160	1	Experienced user	PPL-Hard
29	-0.75	-0.5	5	-0.75	-0.75	165	0.75	Naïve user	PPL-Hard
30	0.75	0	0	2.25	-0.5	110	2	Naïve user	PPL-Hard
31	1	-0.75	75	0.75	-0.75	120	2	Experienced user	PPL-Soft
32	0	-1	173	-0.25	-0.75	2	2	Naïve user	PPL-Hard
33	1.25	-1	90	1	-0.75	80	2	Naïve user	PPL-Hard
34	-0.25	-0.5	90	-0.25	-0.5	95	2.5	Experienced user	PPL-Hard
35	-1.25	-0.75	80	-1.5	-1.25	85	2	Experienced user	PPL-Soft
36	0.25	-0.25	80	0	-0.25	85	1.75	Naïve user	PPL-Hard
37	2	0	0	2.5	0	0	2.5	Naïve user	PPL-Soft
38	2	-0,5	180	1,75	-0,25	120	2	Experienced user	PPL-Soft

### EM behavior depending on user PPL previous experience

No statistical differences between EMs and user PPL previous experience were found. Results of the analysis are shown in Table 2. For each PPL design (PPL-soft and PPL-hard) an analysis was carried out to compare the ocular movement performance between experienced and naïve users

**Table 2.** EM differences according to the subjects’ PPL experience when subjects were using PPL-soft and PPL-hard. Statistical significance p<0.05, randomized complete block test.

When using the PPL-soft	Experienced (Mean ± SD)	Naïve (Mean ± SD)	Df	F-ratio	p-value
Complete fixation time (ms)	9100 ± 500	8600 ± 500	1	0.72	0.38
Number of fixations	29 ± 2	27 ± 1	1	0.66	0.53
Fixation duration mean (ms)	320 ± 10	300 ± 10	1	1.22	0.22
Saccade duration mean (px)	120 ± 30	130 ± 20	1	1.65	0.71
Saccade distance mean (px)	59 ± 3	65 ± 3	1	3.9	0.06
Number of regressions	3 ± 1	4 ± 1	1	3.02	0.07
When using the PPL-hard	Experienced (Mean ± SD)	Naïve (Mean ± SD)	Df	F-ratio	p-value
Complete fixation time (ms)	11000 ± 600	9000 ± 500	1	0.1	0.07
Number of fixations	31 ± 2	28 ± 1	1	0.31	0.08
Fixation duration mean (ms)	340 ± 10	320 ± 10	1	0.95	0.06
Saccade duration mean (px)	64 ± 17	66 ± 15	1	0.18	0.93
Saccade distance mean (px)	51 ± 3	57 ± 3	1	2.02	0.06
Number of regressions	3 ± 1	3 ± 1	1	1.09	0.89

### EM behavior depending on user PPL preference

For each PPL design (PPL-soft and PPL-hard) an analysis was carried out to compare the ocular movement performance between group 1 and group 2. In both cases, using PPL-soft or PPL-hard, group 1 showed different reading pattern than group 2. When participants were using the PPL-hard, complete fixation time, fixation duration mean and number of regressions were statistically significantly lower for group 2 than for group 1, meaning that the time spent on the stops has less duration and less go-back movements influencing reading performance (Table 3).

**Table 3.** EM differences according to the user’s PPL preference when participants were using PPL-soft and PPL-hard. Statistical significance p<0.05, randomized complete block test.

When using the PPL-soft	Group 1	Group 2	Df	F-ratio	p-value	Bonferroni		
	(Mean ± SD)	(Mean ± SD)				Significance	Difference	+/- Limits
Complete fixation time (ms)	8700 ± 3300	7700 ± 2300	1	7,99	0.01*	**	-1054,35	841,84
Number of fixations	27 ± 8	27 ± 7	1	0,41	0.59		-0,64	2,26
Fixation duration mean (ms)	320 ± 70	290 ± 40	1	15,46	0.00*	**	-29,46	16,91
Saccade duration mean (px)	140 ± 110	150± 140	1	0,55	0.52		12,76	38,66
Saccade distance mean (px)	65 ± 15	63 ± 14	1	0,62	0.33		-1,51	4,36
Number of regressions	4 ± 3	4 ± 3	1	0,55	0.30		12,76	38,66
When using the PPL-hard	Group 1	Group 2	Df	F-ratio	p-value	Bonferroni		
	(Mean ± SD)	(Mean ± SD)				Significance	Difference	+/- Limits
Complete fixation time (ms)	8900 ± 3800	7500 ± 2500	1	10,49	0.00*	**	-1325,46	923,53
Number of fixations	28 ± 9	26 ± 7	1	4,8	0.02*		-2,32	2,39
Fixation duration mean (ms)	310 ± 60	290 ± 60	1	6,61	0.01*	**	-19,45	17,07
Saccade duration mean (px)	140 ± 100	130 ± 80	1	0,1	0.78		-3,80	27,05
Saccade distance mean (px)	64 ± 16	64 ± 13	1	0,15	0.78		-0,75	4,42
Number of regressions	4 ± 3	3 ± 2	1	5,49	0.02*	**	-0,77	0,74

## Discussion

The results of this study show that there is a relationship between the EMs of the user and the preference for a specific power distribution of a PPL design. Subjects with lower complete fixation time, lower fixation duration mean and lower number of regressions when reading at a computer prefer a PPL with wider undistorted viewing areas and faster growth of the unwanted astigmatism whilst the participants with higher complete fixation time, higher fixation duration mean, and higher number of regressions prefer a PPL with smoother transitions and a more limited undistorted viewing area. The results also showed how PPL optical performance is affected by the power distribution of the PPL and how the lateral astigmatism and the power of the lens influences the effectiveness of reading.

Eye tracking techniques have been used to evaluate the performance of the lenses while tested by the subjects, and to determine the eye and head movements when using the lenses. In this sense lateral aberrations of PPLs have an important role in the visual behavior and satisfaction of the wearers. Y. Han et al. (18) compared two different PPLs and one single-vision lens on 11 presbyopic participants with normal vision. Six of them were previous PPL users and 5 of them were naïve. Reading at computer was simulated using two hard copy text formats printed in a standardized single page 60 cm away from the subject. Participants had to read out loud the texts and rate their reading ability in 1-7 scale. Eye and head movements were analyzed using ISCAN integrated eye-head movement computer-based system. The results showed a subjective preference for single vision lens against PPLs. In addition to this, most of the EM parameters analyzed were affected by the PPLs in comparison with single-vision lenses. As expected, single-vision lenses provide a better performance when a task at a given distance is performed. When using PPLs the power distribution of the lens has an important effect on reading performance. The power distribution of PPLs determines the lens performance and as discussed, affects the EMs. Hutchings et al. (23) evaluated eye and head movements using an eye tracking system, with two different PPLs. Objective measurements were recorded using an EL-MAR 2020 binocular CCD video eye tracker in 10 participants with no previous experience with PPL. Participants tested two different PPL designs and the results for the head and eye parameters when reading a text placed at 40 cm didn't show differences between PPL designs. Although in the comparison the widths of the different visual areas were given, there was no information about the power distribution, and this also plays an important role in the determination of the satisfaction of the wearer, as shown by Concepción et al. (9). In this pilot study (9), we compared the objective information provided by the eye tracker analysis and correlated it with subjective preference information from subjects. EMs were recorded on 9 presbyopic participants without previous experience using PPLs when they were reading a text on a computer screen using two different PPLs, also collecting the subjective preference for the lens. The technical characteristics of both designs were well-described finding a relationship between final preference and visual skills. The results suggested a different EM performance in PPL designs with lower amount of unwanted astigmatism in the lateral region, which was the lens selected by 90% of the participants. This study was limited because of the low number of participants, that was increased in the research reported here. In the present research, the analysis data was extended including the information from user’s preference and EMs. Similar to our findings, the results suggest that there are two visual profiles differentiated in their visual skills: those participants with lower complete fixation time, lower fixation duration mean and lower number of regressions who tend to prefer a harder PPL design with wide and clear undistorted viewing area, and those participants with higher complete fixation time, higher fixation duration mean and higher number of regressions who tend to choose a softer PPL design, with lower values of lateral astigmatism. Consequently, the results of this study also suggest that eye-tracking systems can be used as part of a PPL design recommendation system according to the user EMs. As power distribution is a key factor on wearer satisfaction, some work has been previously published to predict user adaptability to different lens designs. Alvarez et al. (3) carried out a clinical trial where they examined the adaptability to different PPLs on 47 participants by the measurement of visual tests which considered parameters as phoria and vergence. Alvarez et al. found that the peak velocity of vergence and the rate of change of phoria adaptation was an indicator for PPL acceptance. This is in line with the results of our study but using the objective measurement of EMs and the type of PPLs. However, the previous design and prescription used by each subject was not collected in our study due to the variability of options in the market. In future studies, it could be interesting to include this analysis to determine a possible correlation between the variation of user's prescription and/or previous design used by experienced wearers.

The fact of being an experienced or naïve wearer of PPLs could be a key factor on the characteristics of the EMs. It could be expected that an experienced user might have learnt to restrict head lateral movements, while a naïve subject might use more EMs than head movements which would lead to the use of the distorted peripheral regions. Thus, an analysis between experienced and naïve users was carried out in this study. The results of this analysis suggest that there is no relationship on EMs between experienced and naïve users. Therefore, the preference results obtained in this research do not depend on the subjects’ previous experience using PPLs in the same way than the study done by Y. Han et al. (18) where the statistical analyses comparing the experienced versus naïve PPL wearers didn’t show any statistically significant differences across all the eye and head movement parameters.

The main limitation in this study came from the type of device used for the eye-tracking. Tobii-Pro-X3-120 is a screen-based eye-tracker, it means, it is a stationary device capable of accurately recording EMs regardless of head movements. To use the PPLs in a more realistic way in our study, the subjects were allowed to use them without any head restriction but with the eye tracking system used, the head movement cannot be recorded and compensated. Another limitation of this system is the position of the lens. The lens is located between the eye and the eye tracker cameras, and therefore, the image of the eye recorded by the eye tracker was affected by a prismatic effect. This doesn’t allow to correctly determine the eye position and it could influence the determination of the saccade length and the determination of the position on the text. Also, another limitation of the system is the object distance used, in this case it was only used for intermediate vision but PPLs could have different performances at different object distances. To avoid these limitations, the idea of using wearable eye trackers become important. This kind of eye-trackers incorporate a gyroscope that provides pitch and yaw movements. In this way, EMs can be compensated with head movements obtaining a more realistic vision gaze data. In addition, wearable eye tracker can directly record the position of the eye without prismatic effect as the eye tracker cameras are between the lens and the eyes. It also allows to record different object distances allowing the evaluation of the PPL for all working distances in a dynamic and realistic environment. Another limitation of the study is that the prior worn design was not collected, this could determine the subjects’ EM behavior and preference. Although is difficult to know exactly the power distribution of PPLs on the market it could be interesting in future studies to consider the user’s prior power distribution.

As a conclusion, the results from this study showed how the peripheral ocular motility is affected by the PPL power distribution and the unwanted astigmatism. When a wearer is using the lateral zones of the lens, EM pattern is different than in the central part of the lens due to the unwanted astigmatism of these areas of the PPL. As expected, the EMs improve when the object distance is well matched to the local addition of the lens region the user is looking through. The main result of this study is that while different subjects have different EM pattern and the EMs are not significantly affected by the PPL used, those participants with lower complete fixation time, lower fixation duration mean and lower number of regressions have a stronger preference for harder lens designs compare to those with higher complete fixation time, higher fixation duration mean and higher number of regressions that tend to prefer a softer power distribution. The results of the study can be applied by optometrists and ECPs to determine with an objective test the power distribution that can be best suited for their customers, according to their EMs, and this can be combined with other optical metrics leading to a lens selection that can provide a better adaptation, ergonomics, and performance.

## Ethics and Conflict of Interest

The author(s) declare(s) that the contents of the article are in agreement with the ethics described in http://biblio.unibe.ch/portale/elibrary/BOP/jemr/ethics.html and that there is no conflict of interest regarding the publication of this paper.

## Acknowledgements

This research was supported by IOT. We also thank the anonymous participant and the optometrists in charge of the measurements.
